# Necrotising Myofasciitis of the Lower Limb Secondary to Extra-Peritoneal Rectal Perforation

**DOI:** 10.7759/cureus.28939

**Published:** 2022-09-08

**Authors:** Mohamed A Radhi, Jamie Clements

**Affiliations:** 1 Surgery, Northern Ireland Medical and Dental Training Agency, Belfast, GBR; 2 Plastic Surgery, Ulster Hospital, Belfast, GBR

**Keywords:** lower-limb reconstruction, flexible sigmoidoscopy, rectal perforation, immunocompetent patient with necrotising fasciitis, nectorising myofasciitis

## Abstract

Necrotising fasciitis (NF) is a severe and life-threatening soft tissue infection that often requires extensive debridement and reconstruction. Isolated extra-peritoneal rectal perforations due to trauma, cancer, inflammatory bowel pathology or iatrogenically induced can rarely cause necrotising fasciitis beyond the perineum. Given its rarity, there is a high threshold of suspicion which often leads to late recognition and poor outcome.

We present a case of necrotising myofasciitis of the right lower limb following occult rectal perforation sustained during elective flexible sigmoidoscopy, and augment this case report with a literature review to guide diagnostics, intervention, and recovery. Therefore, the aim of this work was to review, compile, analyse, and present clinical details to identify masquerading presentations and determine the optimal treatment regimen. A search of PubMed, Scopus, Ovid, MEDLINE, EMBASE, CINAHL Plus, AMED, Web of Science (Science Citation Index), and Google Scholar was supplemented by hand searching. Data extracted included demographics, patient management, and outcome.

Of 104 citations identified by a systematic literature search, eight case reports of eight subjects with necrotising fasciitis of the lower limb secondary to rectal perforation met the criteria for analysis. The most common treatment modality was surgical debridement in all cases and bilateral above knee amputation in one case, disarticulation of the lower limb was the treatment in this case we report. Furthermore, faecal diversion by the formation of de-functioning colostomy was performed in the same setting for four (50%) of the patients and appeared to increase survival. Overall 45 days mean (S.E.) disease-specific survival was found to be 32.8 (7.0) days. There is an insufficient number of cases reported to date to confer a significant survival advantage between having a defunctioning colostomy in the same setting as the debridement as opposed to having it at a later setting or not having it at all (Mantel-Cox p=0.1).

In summary, a review of all the cases in the literature suggests that NF of lower limbs can be an atypical presentation of rectal cancer, pathology, and/or trauma. We report a case of unilateral lower limb NF secondary to rectal perforation in a non-cancer patient, likely due to flexible sigmoidoscopy. Due to the small number of patients, it is inherently difficult to draw firm conclusions however multi-modality management appears to be more effective, with meticulous debridement, defunctioning of the bowel and downstaging radiotherapy if required. We recommend a UK-wide, national database/registry for NF that will help gather data and formulate more standardised management guidelines.

## Introduction

Necrotising fasciitis (NF) or flesh-eating disease, is one of the multiple necrotising soft tissue infections (NSTI). It is a rare and dangerous disease with an incidence of 0.4 and 0.53 cases per 100,000 population [1]. The mortality rate of NF ranges from 24% to 34% [2]. Diagnosis of NF is often clinical, manifesting hallmark signs and symptoms including erythema, crepitus, pain out of proportion, lack of bleeding on soft tissue dissection and classic dishwater purulent discharge are hallmarks of this condition. The use of Laboratory Risk Indicator for Necrotising Fasciitis (LRINEC) score was developed by Wong et al. in 2004 to distinguish necrotising fasciitis from severe cellulitis [3]. It is often misdiagnosed and undertreated as many less sinister conditions may masquerade it.

Abdominal visceral perforations are recognised risk factors of NF. Presenting with signs of peritonism help in early recognition and halting progression for NF. However, there is a lack of reported cases of isolated extra-peritoneal rectal perforation causing NF. The absence of peritonism in this unusual pathology makes the abdominal symptoms less pronounced and thereby making the direct etiological reasoning less ubiquitous. Moreover, defunctioning the bowel to reduce the risk of contamination and disease progression is crucial. This is either performed in the same setting as the emergency debridement or undertaken in a delayed fashion, permitting initial source control and stabilisation of the critically unwell patient.

Therefore, the aim of this review is to compile, analyse, and present clinical details of reported cases of NF secondary to extra-peritoneal rectal perforation in order to aid in early recognition and treatment and minimise hazardous outcomes of such a time-ticking condition. In addition to reporting a single patient’s journey at our centre, we are aiming to highlight the different approaches to immediate and intermediate defunctioning of the bowel and explore whether or not there is an effect on patient survival. To the best of our knowledge, this is the first comprehensive review article on lower-limb necrotising fasciitis from extra-peritoneal rectal perforation.

## Case presentation

A 43-year-old male, with a history of diverticular disease and per rectal bleeding presented to the emergency department (ED) of The Royal Victoria Hospital in Belfast with three days history of right lower limb pain and weakness. Of note, he had undergone an elective flexible sigmoidoscopy eight days prior to ED attendance to investigate bright red per rectal bleeding with a flexible sigmoidoscopy under sedation which he reported was very uncomfortable at the time. Physical examination revealed spreading erythema, warm and painful skin with crepitus overlying his right gluteal region, and antero-medial thigh with crepitus. He demonstrated features of sepsis (pyrexia 38 °C, heart rate {HR} 120 beats per minute, blood pressure {BP} 104/68 mm Hg). Abdominal examination including genitalia and the perianal region was unremarkable.

Laboratory findings were as follows (Table 1): haemoglobin 135 mg/dL (135-170 mg/dL), WBC count of 16.4 x 10^9^/L (4-10 x 10^9^/L), C-reactive protein of 458 mg/L (<5 mg/L). Renal function tests revealed a creatinine of 81 micromol/L (62-106 micromol/L), urea of 13.3 mmol/L (2.5-10.7 mmol/L) and sodium of 140 mEq/L (135-145 mEq/L). Liver function tests were within normal limits. Blood gas analysis revealed a metabolic acidosis picture with a pH of 7.29 and lactate of 11.7 mmol/L (0.5-1 mmol/L) and glucose of 4.7 mmol/L.

**Table 1:** Laboratory Findings

Test	Finding	Reference range
Hemoglobin	135 mg/dL	135- 170 mg/dL
White Cell Count	16.4x 10^9^ L	4-10 x10^9^ L
C-Reactive Protein	458 mg/L	<5 mg/L
Creatinine	81 micromol/L	62-106 micromol/L
Urea	13.3 mmol/L	2.5- 10.7 mmol/L
Sodium	140 mEq/L	135-145 mEq/L
pH	7.29	7.35-7.45
Lactate	11.7 mmol/L	0.5-1.0 mmol/L
Glucose	4.7 mmol/L	3.6-5.3 mmol/L

**Figure 1:**
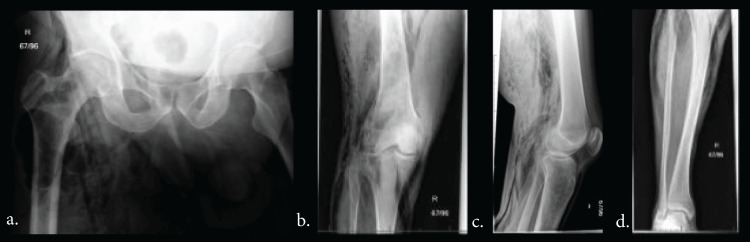
Plain film showing air in tissue planes a. Pelvic X-ray, b. right knee posteroanterior (PA) film, c. right knee lateral film, d. right lower limb PA film.

**Figure 2:**
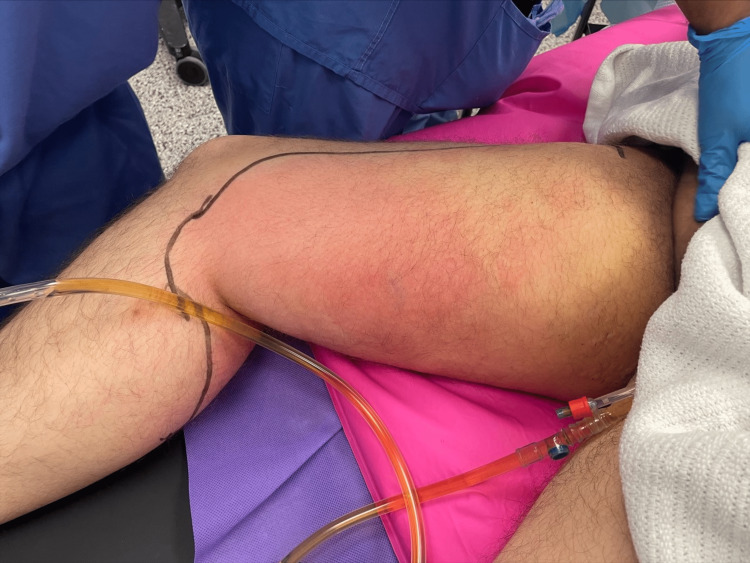
Erythematous edge marked preoperatively Cental palor area was noted, extended from anteromedial to posterior thigh from buttock to posterior calf.

Plain X-rays of the pelvis and lower limbs showed gas in the subcutaneous, fascial and muscular tissue planes (Figure 1). Although the above results yielded an LRINEC score of 5, a high index of suspicion through clinical history and physical examination of spreading cellulitis was used to make a diagnosis of necrotising fasciitis (Figure 2).

Microbiologists were contacted and the patient was started on broad-spectrum antibiotics as per the hospital protocol: piperacillin-tazobactam 4.5 g intravenous (IV) + clindamycin 1.2 g. The patient was immediately transferred to the operation theatre for test incisions and debridement. Test incisions evidenced 'dish-water' fluid and frank necrosis of myofascial tissues in the posterior compartments. Intraoperatively the patient underwent a 3-compartment thigh fasciotomy revealing grey fascia that easily peeled off the underlying devitalised muscle with foul-smelling dishwasher discharge that was sent imminently for microbiology and histopathology respectively. A multidisciplinary (orthopaedic/ plastic/ general surgery) decision on the table was made to proceed with a right hip disarticulation. A myofascial flap was formed from healthy tissues of the anterior thigh compartment and the defect was closed directly. An incisional vacuum-assisted closure (VAC) was placed to aid wound splintage and exudate control and the patient was transferred to a level 3 critical care bed, with a relook planned for the subsequent 24-48 hours.

**Figure 3:**
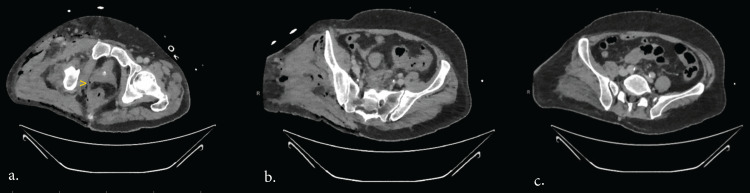
Cross-sectional imaging showing air below the peritoneal reflection Postoperative CT scan revealed the following: (image a) gas around the rectum (yellow arrow) caudally, below the peritoneal reflection. Sigmoid diverticula apparent (image b) without any pneumoperitoneum in more cephalic cross sections (image c).

A computerised tomography (CT) scan of the abdomen pelvis and lower limbs was obtained postoperatively revealing a pattern of gaseous spread that would suggest a perforation below the level of the peritoneal reflection, locules of gas extend inferiorly, tracking along the rectal fascia and presacral region in keeping with a rectal perforation rather a primary sigmoid perforation. In addition to post-operative changes of extensive soft tissue inflammation, intramuscular oedema and a large volume of subcutaneous and intramuscular gas surrounding the right hemipelvis extending inferiorly to the level of amputation (Figure 3).

Histopathological examination showed skeletal muscle which appears non-viable in areas. A mixed inflammatory infiltrate composed mainly of neutrophils. The features were of actively inflamed partially necrotic muscle and while not specific were compatible with necrotising fasciitis. There was no evidence of malignancy. A retrospective review of the sigmoidoscopy report showed extensive moderate congestion and erythema within descending colon; multiple diverticula localised with moderate narrowing/tortuosity of the diverticular segment, mucosal inflammation, and circular muscle hypertrophy within from rectosigmoid junction to the proximal sigmoid colon. Biopsies of the rectosigmoid junction and distal colon revealed brown pigment-laden macrophages in lamina propria in keeping with melanosis coli.

Microbiological specimens of fluid sent intraoperatively grew *Streptococcus anginosus,* *Clostridium sp, *and *Fusobacterium necrophorum*. Tissue cultures grew *E. coli *and *Streptococcus constellatus*. Furthermore, blood cultures grew *Staphylococcus epidermis*, *Staphylococcus capitis, and E. coli*. Antibiotics were rationalised in accordance with the sensitivities of the above microorganisms as per the microbiologist’s advice.

After surgery, the patient remained ventilated and required inotropic support to achieve haemodynamic stability as well as immune globulin intravenous (IVIg) therapy (privigen) in the intensive care unit. Moreover, the patient required a de-functioning colostomy for faecal diversion (two days postoperatively) and two further debridements prior to primary closure. The outcome was a long hospital stay for rehabilitation with the help of tissue viability specialists, physiotherapists, and dietician support for wound care and optimisation of healing. The pressure effect on the residual myocutaneous flap was offset by incisional VAC and air-fluidised mattress to good effect.

Review

Materials and Methods

**Figure 4:**
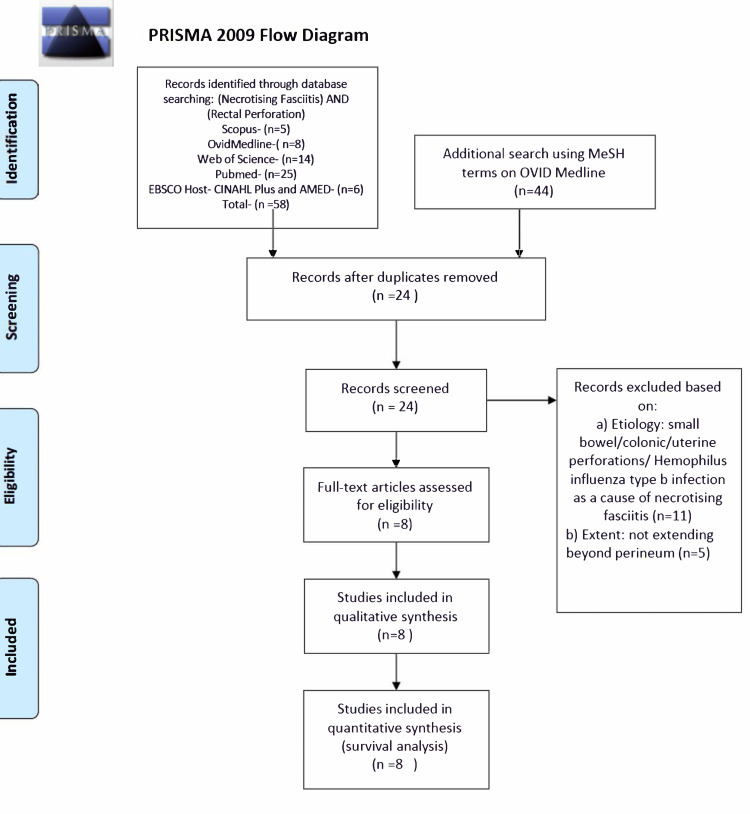
PRISMA Chart PRISMA: Preferred Reporting Items for Systematic Reviews and Meta-Analyses

Search strategy and inclusion criteria: A comprehensive literature search on PubMed, Scopus, Ovid MEDLINE, EMBASE, CINAHL Plus, AMED, and Web of Science (Science Citation Index) for necrotising fasciitis and rectal perforation was performed. Keywords used were necrotising fasciitis, soft tissue infection, rectal perforation, and rectal tear. The search was further extended by using other related MeSH terms like Intestinal Perforation, Necrotizing Fasciitides; Necrotizing Fasciitis; Necrotizing Fascitides and Necrotizing Fascitis. This yielded a total of 104 articles. Articles were reviewed (Figure 4), and duplicates were removed. Publications on necrotising fasciitis secondary to rectal perforation were included. Book chapters and microbiological, antivenom and pharmaceutical studies were excluded. Publications on NF secondary to abdominal visceral perforation not involving the rectum and not extending beyond the pelvis were excluded.

Reference lists from the relevant articles were then inspected and cross-referenced and any other pertinent publications were added to the review. Relevant details of articles in languages other than English were extracted and tabulated by the author separately. A total of eight articles (with eight cases) that satisfied the inclusion criteria, were tabulated, and finally reviewed in this first-of-its-kind study.

Data extraction and analysis: Data on the year of publication, age and gender of the patients was collected. Diagnostic modalities including subjective observations, imaging, microbiology and diagnostic criteria were also noted (Table 2). Treatment given for NF and long-term outcomes were also recorded. Descriptive analysis was performed and mean and median time of disease-specific survival up to 45 days postoperative (with 95% confidence intervals) were calculated.

**Table 2:** Reviewed articles post-op: post-operatively

Article	Patient population	Patient Sex	Age	Diagnostic tool	Microbiology	Management	Survival
Liu et al. (2006) [4]	rectal cancer	Male	56	clinical/ surgical exploration	Group G streptococcus	-bilateral above knee amputation, upper limb debridement, no intra-abdominal/perineal involvement, - defunctioning not performed	died 6 days post op due to multi-organ failure
Highton L (2007) [5]	rectal cancer	Male	79	Plain Xray/ MRI/ surgical exploration	E. coli and anaerobes	-surgical debridement and defunctioning colostomy at a different setting, interval SSGT, interval downstaging radiotherapy and tumour excision	survived, follow-up not mentioned
Fu et al. (2009) [6]	Trauma (self-administered rectal irrigation 2 -cm defect in the left posterolateral wall of the lower rectum.) rectal cancer managed with ultraslow anterior resection and coloanal anastomosis 5 years prior to presentation,	Male	73	CT	Pseudomonas aeruginosa and Enterococcus species	-surgical debridement of the posterior compartment -defunctioning transverse loop colostomy in the same setting	survived able to ambulate independently 2 years post-op
Khalil et al. (2010) [7]	rectal cancer	Male	71	CT	not mentioned	- laparotomy, hartmanns, debridement of the ischiorectal fossa, three compartment fasciotomy of the thigh in the same setting	lung metastasis 30 months after surgery and died 6 years post-op
Park et al. (2012) [8]	rectal cancer -low anterior resection 4 years prior	Male	66	CT/MRI	*Klebsiella pneumonia*, *Enterococcus faecium*, *Acinetobacter baumannii*	-fascial resection of necrotising fasciitis, -ileostomy was offered but the patient refused	died 30 days post-op
Haemers et al.(2013) [9]	rectal cancer	Male	66	CT	E coli, Group Ghaemolytic Streptococcus, and *Candida albicans*	admitted to a surgical ward with the initial diagnosis of erysipelas, then taken to theatre for debridement 24 hrs after admission, defunctioning colostomy planned at a later setting	died 2 days post-op
Evans et al.(2015) [10]	rectal cancer	Male	62	CT	polymicrobial	- surgical debridement - end colostomy in a later setting	died 6 weeks post-presentation
Yang et al. (2019) [11]	rectal cancer	Male	73	CT	not mentioned	Emergency fasciotomy - loop colostomy in the same setting	discharged within 43 days in a stable condition

Results

Since the number of studies published on lower limb necrotizing fasciitis secondary to rectal perforation is minimal, a proper meta-analysis was not possible. Therefore, simple, descriptive statistics have been presented as follows. All eight patients were male. The mean age of the patient population was 68.25 (56-79) years. In the majority (87.5%) aetiology was reported to be secondary to rectal carcinoma; either as the first presentation of rectal carcinoma (Highton et al. [5], Khalil et al. [7], Haemers et al. [9], Evans et al. [10] ), as a result of a perforated rectal carcinoma or as a result of treatment of rectal carcinoma (radiation-induced colitis, Park et al. [8]). Trauma was found to be the cause in one patient following self-irrigation of the rectum (Fu et al. [6]).

An interesting observation was the laterality of the limb involved, 62.5% were NF of the right lower limb, (25%) of the left and (12.5%) bilateral. The most utilised diagnostic modality was a CT scan. Management was aggressive debridement and fascial resection in all cases, in addition to immediate or interval faecal diversion in 50% of cases. This was offered in the form of immediate loop colostomy (2/8), immediate end colostomy (1/8), and defunctioning colostomy scheduled at a later date (3/8).

Notably, all the patients that had a defunctioning colostomy at the time of debridement survived 45 days of follow-up. Hence, a Kaplan-Meier survival analysis was performed for patients that did not receive an immediate defunctioning colostomy (group A) versus patients that received an immediate defunctioning colostomy together with their initial debridement (group B). Overall 45 days mean (S.E.) disease-specific survival was found to be 32.8 (7.0) days (Table 3).

**Table 3:** Means and Medians for Survival Time a. Estimation is limited to the largest survival time if it is censored.

Mean^a^					Median			
Estimate Group e		Std.Error	95% Confidence Interval		Estimate	Std.Error	95% Confidence Interval	
			Lower Bound	Upper Bound			Lower Bound	Upper Bound
A	20.750	8.813	3.476	38.024	6.000	14.000	0.000	33.440
B	45.000	.000	45.000	45.000	.	.	.	.
Overall	32.875	7.099	18.961	46.789	45.000	.	.	.

There is an insufficient number of cases reported to date to confer a significant survival advantage between having a defunctioning colostomy in the same sitting as the debridement as opposed to having it at and later setting or not at all (Mantel-Cox p=0.1) (Table 4).

**Table 4:** Overall Comparisons df: Degrees of freedom

Chi-Square		df	Sig.
Log Rank (Mantel-Cox)	2.697	1	0.101
Breslow (Generalized Wilcoxon)	3.025	1	0.082
Tarone-Ware	2.891	1	0.089

## Discussion

Due to the rarity of necrotising fasciitis, there are no ongoing surveillance programmes that capture data on NF cases [12]. An estimated 500 cases present each year in the UK [13]. To the best of our knowledge, this is the first case reported of lower limb NF secondary to completely extraperitoneal rectal perforation in a non-rectal cancer patient. It is difficult to ascertain whether this was an iatrogenic perforation secondary to flexible sigmoidoscopy eight days prior to presentation, as a CT scan was not done prior to debridement. The diagnosis was made based on plain radiographic findings and a clinical picture.

Although the post-debridement CT scan showed gas below the level of the peritoneal reflection in keeping with rectal perforation; the perforation may have been due to inadvertent debridement of devitalized gluteal tissue. Identifying the source or aetiology of NF preoperatively may delay the patient’s definitive surgical management and, in our case, did not affect the outcome. It is well established that treatment should not be delayed regardless of how sophisticated the diagnostic modalities available i.e. ‘time is muscle’.On the contrary, the cases included in this review, apart from Liu et al. [4], all utilised CT to aid in diagnosis. That is likely due to the challenging nature of diagnosing NF.

Diagnosing NF is puzzling, a meta-analysis by Fernando et al. (2019) [14] illustrated the following signs and symptoms having the respective sensitivity and specificity: haemorrhagic bullae 25.2% and 95.8%, and hypotension 21.0% and 97.7%. Computed tomography (CT) has a sensitivity of 88.5% and a specificity of 93.3%, while plain radiography had a sensitivity of 48.9% and a specificity of 94.0%. Finally, LRINEC ≥ 6 had a sensitivity of 68.2% and specificity of 84.8%, while LRINEC ≥ 8 had a sensitivity of 40.8%and specificity of 94.9%.

NF can be classified into monomicrobial (type I), polymicrobial (type II), gas gangrene caused by Clostridium species/Vibrio species/Aeromonas (type III), and due to fungal infections in immunocompromised patients (type IV [15]. Type II is the most common, as it was in the case we are reporting. Similarly, 87.5% of cases included in this review were type II, apart from Liu et al. (2006) [4]-Group B streptococcus.

Treatment of NF revolves around aggressive debridement of devitalised tissue. Which often leaves sizeable soft tissue defects requiring interval reconstruction. Cases included in this comprehensive review did not delve into details of reconstruction, apart from Highton et al. (2007) [5]-interval split-thickness skin graft. However, a remarkable discrepancy in approaching bowel defunctioning was noted, which was highlighted in our descriptive analysis. This could be due to the ambiguity of aetiology which requires case-by-case tailored management. The reasoning behind interval faecal diversion was only mentioned in Highton et al. (2007) [5]-rectal tumour required down staging radiotherapy and interval excision.

The prognosis of NF is poor with disastrous outcomes. In our case, the patient required a long-term hospital stay, rehabilitation, a pressure relieving mattress, and input from multiple disciplinaries. Right hip disarticulation left the patient with poor functional outcomes. Similarly, the cases included in this review most had an occult presentation and a delayed diagnosis with the aid of a CT scan in six out of eight patients.

## Conclusions

In summary, we report a case of unilateral lower limb necrotising myofasciitis secondary to extraperitoneal rectal perforation. This was treated with right lower limb disarticulation, defunctioning colostomy, and required multiple reconstructive procedures. In conjunction with the case reported, a review of the limited relevant literature has evidenced that NF of the lower limbs is an exquisitely rare complication of malignant and benign rectal pathologies and/or trauma. In many cases, this is a life-limiting complication and in all instances life-changing, with limb loss, intestinal diversion, and prolonged hospital admissions universally described. Due to the small number of patients, it is inherently difficult to draw firm conclusions, however, multi-modality management appears to be more effective, with meticulous and early debridement, defunctioning of the bowel, and downstaging radiotherapy if required. We recommend a UK-wide, national database/registry for NF that will help gather data and formulate more standardized management guidelines.
